# MCATs: Case report of adnexal adenocarcinoma of not otherwise specified type

**DOI:** 10.1002/ccr3.7097

**Published:** 2023-03-15

**Authors:** Tetyana Svyatenko, Andrii Prokhach, Stepan Antoniuk, Vadym Hurtovyy, Denys Tereshkov, Anna Prokhach

**Affiliations:** ^1^ Dnipro State Medical University Dnipro Ukraine; ^2^ Dnipro Regional Pathological Bureau Dnipro Ukraine; ^3^ City Clinical Hospital №4 Dnipro Ukraine; ^4^ Clinic of Prof. Svyatenko Dnipro Ukraine

**Keywords:** adnexal adenocarcinoma, adnexal skin tumors, MCAT's, rare cutaneous neoplasia

## Abstract

Malignant cutaneous adnexal tumors are rare dermatosis that can have extradermal twins. Diagnosis of adnexal skin tumors is sometimes difficult. Immunohistochemistry has limited value in the diagnosis. Most adnexal skin tumors are localized on the head and neck. Difficulties in their diagnosis associated with absence of strict clinical criteria.

## 1 INTRODUCTION

Adnexal skin tumors are a group of rare, heterogeneous benign and malignant tumors with possible apocrine, eccrine, follicular, and multilinear differentiation. Some of the adnexal tumors have extradermal twins, typical of the salivary and mammary glands, but most are unique to the skin. Benign neoplasms are more common than malignant and can occur at any age. Carcinomas are rarer, and their episodes increase with population aging. Adnexal tumors have a wide variety of clinical manifestations: They can grow in the form of solitary formations and multiple papules, nodules, plaques. Malignant cutaneous adnexal tumors (MCATs) usually appear de novo, but some may occur on the background of previous benign lesions. Diagnosis of adnexal skin tumors is sometimes difficult, due to their ability to form cysts, to have pigmented variants. Immunohistochemistry has limited value in the diagnosis of MCATs but in some cases may provide significant data. Most adnexal skin tumors are localized on the head and neck, where they can mimic other tumors. Adnexal adenocarcinoma of not otherwise specified type (NOS) is a primary adenocarcinoma of the skin with ductal/glandular type of differentiation but without specific histological features for further classification.^1^

## 2 CASE REPORT

**FIGURE 1:**
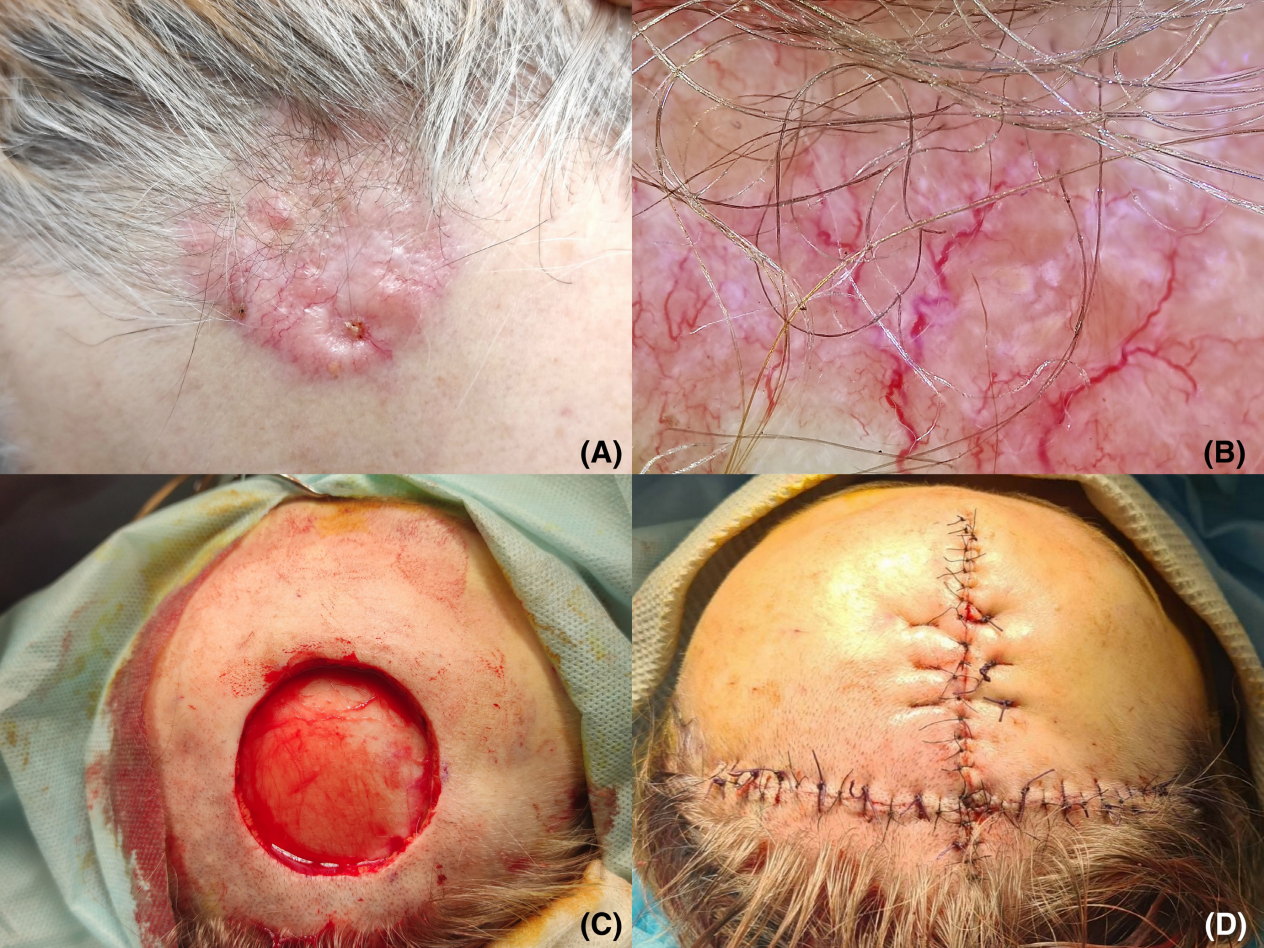
Adnexal adenocarcinoma of the skin: A—lesion on the upper forehead; B—dermoscopic features: serpentine‐like vessels, linear telangiectasias, “rainbow” pattern; C—defect after tumor excision; D—«A‐T» wound closure.

**FIGURE 2:**
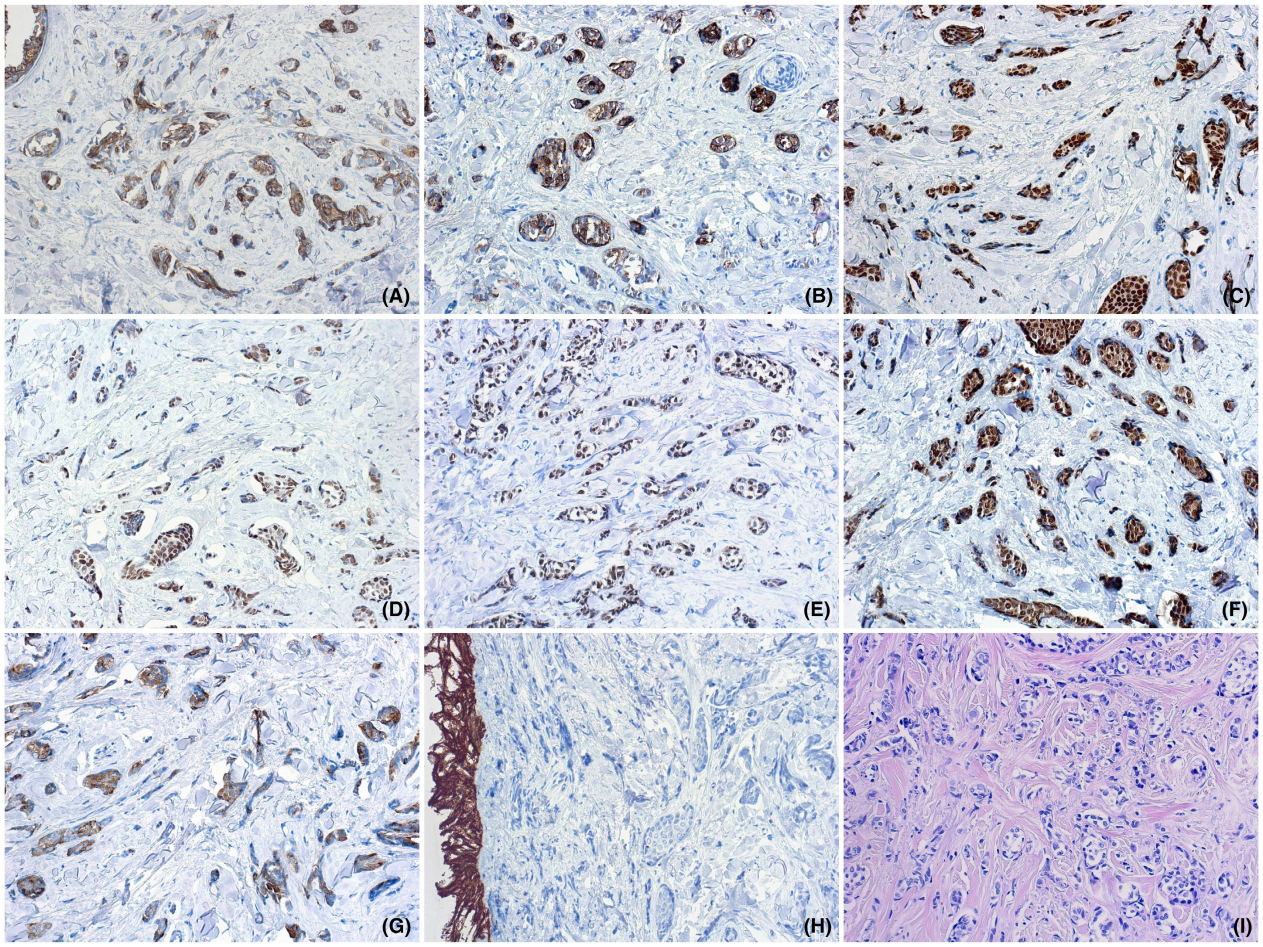
Immunohistochemical profile of the tumor: a—membrane expression of tumor cells to CK7, ×200; b—positive cytoplasmic reaction of cells to GCDFP 15, ×200 antibodies; c—the majority of tumor cells demonstrate a pronounced nuclear expression to antibodies GATA‐3, ×200; d—weak nuclear expression of p40, ×200 cells; e—the tumor is positive for ER receptors, ×200; f—the tumor is positive for PgR receptors, ×200; g—positive membrane expression of tumor cells to mammoglobin, ×200; h—negative membrane expression of tumor cells to CK5, ×200; i—pronounced infiltrative tumor growth with the formation of cribriform, nested and trabecular structures, hematoxylin–eosin, ×200.

**FIGURE 3:**
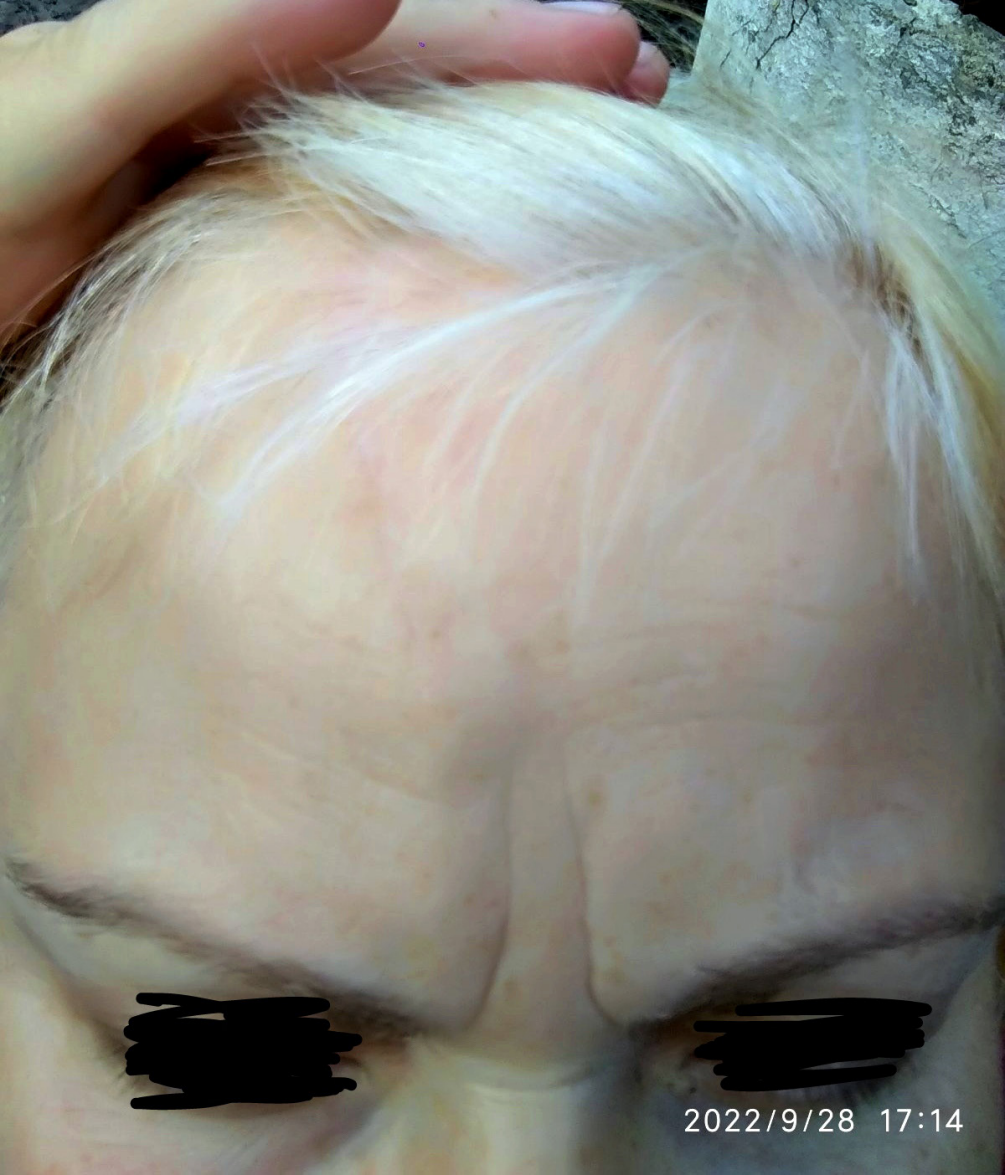
Condition 6 months after surgical treatment.

We observed a 53‐year‐old woman with lesion on the right forehead, who had been ill for the past 4 years. Destruction procedures were repeatedly performed, which did not lead to complete removal of the formation. Clinically and dermoscopically there defined erythematous plaque of dense consistency, 25 × 25 mm with the presence of linear and serpentine telangiectatic vessels, erosions and “rainbow” pattern (Figure 1).^2^ In order to verify, an incisional biopsy was performed. On histological examination, the tumor extends to the entire depth of the dermis. The epidermis over the tumor is thinned. A pronounced infiltrative growth of atypical tumor cells with the formation of nests, trabeculae, and cribriform structures is noted. Part of tumor cells with polymorphic, angular nuclei and coarse chromatin. The nuclei of cells that form solid nestedstructures are round or oval with lightened delicate chromatin; the cytoplasm is cleared. There is a marked desmoplastic reaction of the stroma around the tumor complexes. There are single mitoses. The lymphoid cell reaction of the stroma is weak and focal. To clarify the histogenesis of the tumor and determine the immunophenotype of tumor cells, an immunohistochemical study was performed (Figure 2). Also, the reason for additional immunohistochemical studies was the frequent metastasis of malignant tumors of other localizations into the skin.^3^ When conducting an immunohistochemical study, most of the tumor cells were positive for the following markers—CK7, GATA 3 (L50‐853), GCDSP‐15, mammaglobin A&B and negative for CK 5/6. GATA3 target gene promoters are involved in epidermal differentiation, and in the skin barrier function, as well as in the differentiation of the mammary glands and the urothelial tract and the regulation of T‐cell differentiation.^4^ Normal apocrine glands could demonstrated a high level expression of GATA3. GCDFP‐15 is reported to be of cutaneous or mammary origin. In addition, tumor cells showed a positive reaction of varying degrees of intensity to ER and PgR receptors.^3^ Since the histological picture, as well as the immunophenotype of the tumor, was similar to non‐specific type ductal carcinoma of the breast,^5^ additional targeted clinical studies of mammary lesions were carried out. Considering all the data we diagnosed adnexal adenocarcinoma of not otherwise specified type.^6^ MCATs are rare neoplasms that do not have a well‐studied treatment algorithm.^7^ According to the risk of recurrence and metastasis, we have discussed the case at multidisciplinary commission. At the first stage of treatment, we used wide excision with «A‐T» wound closure^8^ and sentinel lymph node biopsy (SLNB).^9^, ^10^ Based on the results of SLNB, the lymph node was not affected. After healing (Figure 3) as the adjuvant antiestrogen therapy was used.^11^

## 3 DISCUSSION

Malignant cutaneous adnexal tumors are rare neoplasms of skin appendages. Difficulties in their diagnosis associated with absence of strict clinical criteria, dermoscopic features. Although most of them show a variety of morphological characteristics, the plastic features of the structure inherent in adnexal tumors are determined. Even pathology that plays big role in diagnostic of skin disease could lead to the diagnosis pitfall. Another problem is related to lack of proven treatment algorithm: Frequency of MCATs does not allow to provide it. They are generally treated by excision alone. The need for SLNB and adjuvant therapy is widely discussed in the literature. That is why it is so important to highlight such cases.

## AUTHOR CONTRIBUTIONS

**Tetyana Svyatenko:** Project administration. **Andrii Prokhach:** Investigation; writing – original draft. **Stepan Antoniuk:** Methodology. **Vadym Hurtovyy:** Methodology. **Denys Tereshkov:** Formal analysis. **Anna Prokhach:** Methodology.

## FUNDING INFORMATION

None.

## CONFLICT OF INTEREST STATEMENT

The authors have no conflict of interest.

## CONSENT

Written informed consent was obtained from the patient to publish this report in accordance with the journal's patient consent policy.

## Data Availability

The data that supports the findings of this study are available in the supplementary material of this article.
